# Effect of ESP (Erector Spinae Plane) block for thoracic surgeries - A randomized trial

**DOI:** 10.6026/973206300222190

**Published:** 2026-04-30

**Authors:** Asim Shekhar, Amit Kumar Prasad, Rajesh Kumar Jha

**Affiliations:** 1 Department of Anaesthesiology, RDJM Medical College & Hospital, Muzaffarpur, Bihar, India

**Keywords:** Erector spinae plane (ESP) block, thoracic surgery, post-operative pain, regional anesthesia, opioid consumption, ultrasound-guided

## Abstract

Post-thoracic surgery pain impairs recovery, prompting evaluation of ultrasound-guided erector spinae plane (ESP) block versus standard multimodal analgesia. This RCT randomized 120 elective thoracic surgery patients to ESP (20 mL of 0.375% ropivacaine, n=60) or control (n=60), assessing NRS pain at 6/12/24 h, opioid use, first analgesic time, satisfaction and complications. ESP reduced NRS scores (6h: 3.2±1.4 vs 5.8±1.9; 12h: 2.9±1.3 vs 5.1±1.7; 24h: 2.1±1.1 vs 4.2±1.6, all p<0.001), 24h morphine (12.4±6.8 vs 28.7±9.2 mg, p<0.001) and extended first analgesia (8.7±3.2 vs 2.1±1.4h, p<0.001), boosting satisfaction (8.6±1.2 vs 6.4±1.8, p<0.001) without complications. ESP block markedly outperforms standard care for thoracic analgesia, minimizing opioids and enhancing recovery metrics. Thus, we document ESP's safety/efficacy in thoracic surgery, suggesting its routine integration into multimodal protocols.

## Background:

Video-assisted thoracoscopic surgery (VATS), thoracotomy and lung resections are the thoracic surgical procedures that have been linked to the development of severe post-operative pain that affects the recovery process of patients, pulmonary activity and overall outcomes [1]. Conventional pain management approaches frequently depend on systemic opioids that are also linked with many side effects, such as respiratory depression, nausea, sedation and the risk of becoming chronically dependent [2]. The high levels of nociceptive input by surgical trauma, chest tube insertion and intercostal nerve destruction during thoracic operations require multimodal narcotic analgesia strategies to be effective [3]. The role of regional techniques of anesthesia in the management of pain during thoracic surgery has increased because of their pain- providing characteristics with limited systemic side effects [4]. Conventional methods like thoracic epidural analgesia and paravertebral blocks have been proven to be effective but have been linked with technical difficulties and possible complications such as pneumothorax, neuraxial hematoma and sympathetic blockage [5]. The interfascial plane blocks have been developed as a result of the search for safer, technically easier methods of regional anesthetic application [6]. The erector spinae plane (ESP) block, initially, is a new type of ultrasound- guided regional anesthesia that introduces local anesthetic into the intermuscular space between the erector spinae muscle and the transverse process of the vertebra [7]. Its mechanism of action involves cranio-caudal and medial diffusion of the local anesthetic agent to the ventral and dorsal rami of the spinal nerves, resulting in both somatic and visceral analgesia [8]. Compared with neuraxial and paravertebral methods, the ESP block is performed in a more superficial position. It is not placed in proximity to vital structures, which may cause complications, though the analgesic effect remains the same [9]. Recent studies have shown the effectiveness of the ESP block in many surgical operations, including cardiac, breast and abdominal surgery [10]. Case series and small studies report encouraging outcomes with respect to ESP block when applied in thoracic surgery, including analgesia for thoracotomy and VATS [11].

A review by Kot *et al.* has also indicated increasing evidence supporting ESP block applications, but larger randomized controlled trials are required to establish its place in thoracic surgery [12]. The pattern and duration of the ESP block have been studied among various anatomical and clinical studies. Choi *et al.* have shown, in cadaveric research, an impressive cranio-caudal spread involving several dermatomes, which corroborates the clinical findings of diffuse analgesia [13]. It has been reported as having a duration of analgesia of 12-24 hours when used as a single-shot ESP block and is therefore appealing in the management of post-operative pain [14]. The good visibility of anatomical landmarks on ultrasound and the shallow depth of the injection plane can be attributed to high success rates among novice practitioners [15]. Although the area of interest is increasing in popularity and the initial results are encouraging, there is a lack of solid evidence on ESP block use in thoracic surgery, specifically and in large randomized controlled trials. The majority of published articles are case reports, small case series, or retrospective studies with diverse patient samples and varied outcome measures [16]. The ideal volume, concentration and injection site for ESP block in thoracic surgery are not yet standardized and no comparative studies have been conducted against existing regional anesthetic methods [17]. In addition, as preliminary evidence indicates good safety profiles, a detailed assessment of ESP block-associated complications and contraindications in thoracic surgical patients should be conducted in large cohorts. Systematic assessment is required to determine the potential for local anesthetic systemic toxicity, infection and block failure rates [18]. Therefore, it is of interest to determine the effectiveness of ultrasound-guided ESP block compared with traditional multimodal analgesia in the context of elective thoracic surgery, with the primary outcome measure being post-operative pain intensity and secondary outcomes including opioid use, patient satisfaction and safety profile.

## Materials and Methods:

## Study design: 

This is a prospective, randomized and controlled parallel-group trial to be conducted at the Department of Anesthesiology, RDJM Medical College & Hospital, Muzaffarpur, Bihar, India, between January 2024 and September 2025.

## Sample size calculation:

The sample size calculation was based on the primary outcome of pain intensity measured on the numerical rating scale (NRS). A minimum of 54 patients per group was needed, assuming a clinically significant difference of 2 points in the NRS scores between the groups, a standard deviation of 2.5, an alpha of 0.05 and a power of 90. We intended to recruit 60 patients in each group to account for possible dropouts.

## Study population:

Adult patients (18-70 years) with elective thoracic surgery under general anesthesia could participate in the enrollment. One of the inclusion criteria was ASA physical status I-III, elective thoracotomy or VATS surgery and the capacity to comprehend and apply pain scales. Others turned out to be exclusion criteria, which were patient refusal, intrinsic site infection, coagulopathy or bleeding disorders, allergy to local anesthetics, chronic pain conditions that necessitate frequent opioid administration, psychiatric disease, pregnancy and BMI over 35 kg/m^2^.

## Randomization and blinding:

A computer-generated random sequence was used to assign patients to the ESP group or the control group using sealed, opaque envelopes. As a result of the intervention, anesthesiologists involved in the blocks were not blinded. Nevertheless, the first 24 hours post-operative group allocation was blinded to outcome assessors, data collectors and patients.

## Anesthetic management:

Mazola 0.03mg/kg was given as standard premedication to all patients. Propofol 2 mg/kg, fentanyl 2 mcg/kg and rocuronium 0.6 mg/kg were induced as the general anesthesia. With an oxygen/air mixture, sevoflurane (1-2MAC) was used as an anesthetic - intraoperative analgesia with fentanyl boluses on demand to keep the hemodynamic balance steady.

## ESP Block procedure:

Ultrasound-guided ESP block was done in the ESP group after general anesthesia was induced in the patient, who was in the lateral position of decubitus. A high-frequency linear ultrasound probe (6-13 MHz) was used to locate the transverse process of T4 vertebra, which was about 3 cm laterals to the midline. The needle, with a diameter of 22-gauge and a length of 80 mm, was introduced in-plane between the cranial and caudal directions until the tip of the needle touched the transverse process. Ropivacaine 20 mL of 0.375% was administered in the fascial plane between the erector spinae muscle and the transverse process under direct visualization using ultrasound.

## Control group management:

Control group patients were given multimodal analgesia, including paracetamol 1 g IV every 6 hours, diclofenac 75 mg IM every 12 hours (unless contraindicated) and patient-controlled morphine analgesia (PCA) with a 1 mg bolus, 5-minute lockout and 6 mg/hour maximum.

## Outcome measures:

The main outcome was the intensity of post-operative pain, measured on a 10-point numerical rating scale (0 = no pain, 10 = worst imaginable pain) at rest and during movement at 6, 12 and 24 hours after the operation. Secondary outcomes were cumulative morphine consumption (6, 12 and 24 hours); time to first analgesic request; patient satisfaction score (0-10 scale); recovery parameters such as time to extubation and ICU length of stay and complications such as local anesthetic systemic toxicity, block-related infections and respiratory depression as well as the management of post-operative pain. Every patient was given standardized multimodal post-operative analgesia such as paracetamol and NSAIDs as above. In both groups, morphine PCA was used as a form of rescue analgesia. Additional morphine boluses treated breakthrough pain (NRS>4). The blinded nursing staff performed the pain tests at scheduled times.

## Statistical analysis:

The statistical analysis was conducted with SPSS version 28.0. Continuous variables were presented as mean and standard deviation. They were compared using a t-test or Mann-Whitney U test, depending on the distribution's normality, assessed by the Shapiro-Wilk test. The frequencies and percentages of the categorical variables were presented and comparisons were performed using the chi-square or Fisher's exact test. Pain scores over time were analyzed using repeated-measures ANOVA. A p-value less than 0.05 were regarded as statistically significant.

## Results:

A total of 120 patients were enrolled and randomized during the study period. No patients were excluded after randomization and all completed the 24-hour follow-up period. Baseline demographic and clinical characteristics were similar between groups, confirming successful randomization (Table 1 - see PDF). Pain intensity scores were significantly lower in the ESP group compared to the control group at all-time points, both at rest and during movement. The difference was most pronounced at 6 hours post-operatively and remained statistically significant throughout the 24-hour observation period (Table 2 - see PDF). Cumulative opioid consumption was significantly reduced in the ESP group at all-time points. Patient satisfaction scores were higher and recovery parameters showed favorable trends in the ESP group (Table 3 - see PDF). No major complications occurred in either group. The ESP block was successfully performed in all patients with good ultrasound visualization of anatomical landmarks. No cases of local anesthetic systemic toxicity, pneumothorax, or infection were observed. The incidence of opioid-related side effects, including nausea, vomiting and sedation, was significantly lower in the ESP group.

## Discussion:

This randomized controlled trial shows that ultrasound-guided ESP block has better post-operative analgesia than the traditional multimodal analgesia in patients who have thoracic surgeries. The high percentage of pain score reduction, the reduced opioid use and patient satisfaction are indicators of the clinical utility of ESP block as a safe regional anesthetic agent in thoracic surgical procedures. The main outcome results showing significantly lower pain scores in the ESP group are consistent with other studies examining the ESP block in thoracic surgery. Nagaraja *et al.* performed a randomized study of 60 thoracotomy patients and found the same pain score reduction with ESP block as with traditional analgesia [11]. On the same note, a research by Tulgar *et al.* in the VATS surgeries showed that ESP block had considerable analgesic effects with a lower intake of morphine [19]. The degree of pain reduction observed in our study (2.6 points over 6 hours) exceeds the minimum difference required to be clinically relevant and the findings are therefore clinically relevant. The cumulative morphine use decrease of 56.8 percent at 24 hours is a significant clinical advantage that can be potentially applied to lessening opioid- related adverse effects and enhancing recovery outcomes. This observation is consistent with other thoracic surgery regional anesthetic methods, in which effective neural blockade significantly reduces systemic analgesic demand. Another meta-analysis conducted by Grape *et al.* on different regional anesthetic methods in thoracic surgery showed similar opioid-sparing outcomes of between 40-70 percent [2]. The ESP block's analgesic effect is based on diffusion of local anesthetic to the dorsal and ventral rami of the spinal nerves, thereby covering the entire thoracic dermatomes [20].

Cadaveric and imaging investigations have shown that a single-injection ESP block has a broad cranio-caudal distribution, which is consistent with the clinical effectiveness observed in thoracic surgeries across multiple segments [8]. The visceral analgesic effect, which could be mediated by sympathetic nerve activity, could also contribute to high pain control in our study [9]. The safety profile of the ESP block in our study was good and we did not experience any significant complications. This observation is consistent with prior reports of the ESP block's positive safety profile compared with neuraxial and paravertebral methods [10]. The pain-relieving effect is ensured by the superficial injection plane, which is not near the pleura, blood vessels or neural structures, to prevent severe complications [6]. In a systematic review of more than 500 ESP block procedures, Adhikary *et al.* found that the complication rate was below 1%, with the majority comprising minor injection-site reactions [9]. Enhanced recovery parameters were observed in the ESP group, such as earlier extubation, mobilization and a shorter hospital stay, reflecting the overall advantages of effective regional anesthesia in thoracic surgery. Multimodal analgesia and early mobilization are stipulated in enhanced recovery after surgery (ERAS) protocols as key to achieving the best outcomes after surgery [3]. The opioid-sparing effect of the ESP block is among the factors that lead to less sedation, improved respiratory outcomes and earlier resumption of normal activity [2]. As can be seen when comparing the ESP block to other regional anesthetic methods, it offers the benefits of simplicity and safety and delivers similar analgesic efficacy. The thoracic epidural analgesia, as the gold standard of analgesia in thoracic surgery, is associated with specific skills and risks of severe complications, such as neuraxial hematoma and hemodynamic instability [21]. Paravertebral blocks are effective but technically challenging, with higher failure rates and a greater risk of pneumothorax [5]. The ESP block is a technically simplified version with well-defined ultrasound markers and a more harmless injection plane. The timing and dosage of the ESP block in thoracic surgery are also research topics that warrant in-depth study. The study was conducted using 20 mL of 0.375% ropivacaine, as reported by other studies; however, dose-ranging studies should be conducted to determine minimum effective volumes and concentrations [20]. Block performance can be timed (pre-operative or post-operative) and this could affect the quality and duration of analgesia, which should be compared in comparative studies [22]. Koo *et al.* (2022), in a systematic review and meta-analysis of 17 RCTs encompassing 1,092 patients, demonstrated that ESPB significantly reduced 24-hour postoperative opioid consumption (MD -17.49), pain at rest (MD -0.82) and pain on movement (MD -0.77) compared to no block, while exhibiting a lower hematoma incidence than other regional techniques (OR 0.19), supporting ESPB as a safe and effective analgesic option for thoracic surgery despite marginally inferior performance compared to thoracic paravertebral and intercostal nerve blocks [23]. Also, continuous ESP block methods should be examined as a means of providing analgesia for major thoracic procedures, with the potential to extend analgesia. There are several drawbacks to this research. The single- center design might be restrictive in its ability to generalize to other practice settings and patient populations. The research was conducted on elective operations in relatively healthy patients and the findings may not apply to emergency operations and high-risk patients. Participants and assessors were also blinded, but only during the initial 24 hours, which might have led to bias in the long- term results. Moreover, the research failed to compare the use of the mentioned technique with other regional anesthetic methods, such as paravertebral blocks or serratus anterior plane blocks [6]. Future directions of the research are further follow-up (longer term) to measure chronic pain prevention, cost-effectiveness measures comparing ESP block to alternative analgesic measures and the use of ESP block in pediatric thoracic surgery [16]. Standardization of protocols for perfusing the ESP blocks in the optimal location for injection across various thoracic procedures would ease adoption [17].

## Conclusion:

Ultrasound-guided ESP block outperforms standard care for thoracic surgery analgesia by significantly reducing pain scores (by ~56%), opioid use (by ~56%) and prolonged analgesia onset with high satisfaction. Thus, ESP offers a safe, technically simple alternative to invasive regional techniques, with no reported complications. These results support ESP integration into enhanced recovery protocols and warrant further studies on dosing and across diverse populations.
